# CTL Attenuation Regulated by PS1 in Cancer-Associated Fibroblast

**DOI:** 10.3389/fimmu.2020.00999

**Published:** 2020-06-10

**Authors:** Hongyu Zhang, Rong Jiang, Jinhua Zhou, Juan Wang, Yuejuan Xu, He Zhang, Yanzheng Gu, Fengqing Fu, Yu Shen, Guangbo Zhang, Lanlan Feng, Xueguang Zhang, Youguo Chen, Fangrong Shen

**Affiliations:** ^1^Department of Obstetrics and Gynecology, The First Affiliated Hospital of Soochow University, Suzhou, China; ^2^Jiangsu Institute of Clinical Immunology, The First Affiliated Hospital of Soochow University, Suzhou, China; ^3^Jiangsu Key Laboratory of Clinical Immunology, Soochow University, Suzhou, China; ^4^Jiangsu Key Laboratory of Gastrointestinal Tumor Immunology, The First Affiliated Hospital of Soochow University, Suzhou, China; ^5^Department of Gynecology, The Second People's Hospital of Taizhou, Taizhou, China

**Keywords:** tumor microenvironment, cancer-associated fibroblasts, immunosuppression, PS1, IL-1β, WNT/β-catenin pathway

## Abstract

**Objective:** Cancer-associated fibroblasts (CAFs) were associated with tumor progression in the tumor microenvironment (TME). However, their immunosuppressive roles in protecting cancer cells from the attack by cytotoxic T lymphocytes (CTLs) are not fully clear. In this study, we investigated whether and how CAFs regulate tumor-infiltrating lymphocytes as well as their role in tumor immunosuppression.

**Methods:** Eighty-three cases of ovarian cancer and 10 controls were analyzed for CAFs and CD8+ tumor-infiltrating lymphocytes by gene array and immunohistochemistry. We evaluated presenilin 1 (PS1) expression in CAFs, CTL penetration, tumor burden, dendritic cell function, and migration of tumor-infiltrating lymphocytes and their function *in vivo* and *in vitro* after silencing PS1. In addition, the pathway via which PS1 affects the TME was also evaluated.

**Results:** PS1 was highly expressed in CAFs, and its silencing significantly promoted CD8+ CTL proliferation and penetration in multiple ovarian models (*p* < 0.05), resulting in tumor regression and growth inhibition. Interleukin (IL)-1β was identified as a major immune inhibitor in the TME, and it was significantly decreased after PS1 silencing (*p* < 0.05), which was regulated by the WNT/β-catenin pathway. It was also showed that high expression of IL-1β in CAFs inhibits CTL penetration significantly (*p* < 0.05).

**Conclusion:** Highly expressed PS1 in CAFs plays a crucial role in regulating tumor-infiltrating lymphocyte populations in the TME via the WNT/β-catenin pathway. Targeting PS1 may retrieve functional CTLs in the TME and improve the efficacy of current immunotherapies.

## Introduction

Immunotherapy is recognized as an important therapeutic strategy for cancers and attracts more and more attention. High levels of intratumoral T cells are strongly and consistently correlated with patient survival in multiple cancer types, including high-grade serous ovarian carcinoma (HGSC) and pancreatic ductal adenocarcinoma (1, 2). However, while immune checkpoint blockade antibodies, such as those against programmed cell death-1 (PD-1) or cytotoxic T-lymphocyte-associated protein 4 (CTLA-4), have shown remarkable efficacy in certain cancer types such as melanoma and non-small-cell lung carcinoma (3, 4), limited therapeutic benefit has been also observed in other types of cancers such as colorectal cancer, ovarian cancer, and pancreatic ductal adenocarcinoma. Indeed, a recent clinical trial of PD-L1 showed limited benefit for ovarian cancer patients, with only 1 out of 17 patients having a partial response (5). Similarly, immunotherapy has, thus far, shown limited efficiency in pancreatic ductal adenocarcinoma. The difficulty of inducing an effective antitumor immune response largely stems from the highly immunosuppressive microenvironment present in these tumors (6).

Cancer cells can regulate and activate a variety of cellular components in the surrounding interstitial environment and express membrane-bound negative regulatory molecules to form a tumor-growth-conducive environment (7). Among them, cancer-associated fibroblasts (CAFs) are the most important components for adhesion support of tumor cells. They can promote the invasion and metastasis of tumor cells by secreting lysyl oxidase to modify the extracellular matrix (8). It has been reported that increased CAF numbers in the extracellular matrix of pancreatic ductal adenocarcinoma significantly reduced the infiltration of CD8^+^ T cells and the therapeutic effect of anti-PD-L1 antibody (2). Previous research has confirmed that CAFs can compete with CXCR4 by secreting CXCL12 to isolate T cells from contact with tumor cells and promote immune escape (9). *In vitro* and *in vivo* experiments have found that CAFs can produce a variety of inflammatory cytokines and chemokines and regulate various immune cell subpopulations in the TME (10). These studies suggest that CAFs may play an important role in tumor immune escape by recruiting immune cells and regulating immune cell functions. Moreover, CAFs are well-known for their immunosuppressive activity as well as their emerging role as a major barrier for cytotoxic T lymphocytes (CTLs) at the tumor site (11, 12). However, whether and how CAFs affect the function and infiltration of CD8^+^ T cells have not been extensively studied yet.

Therefore, in this study, we hypothesized that CAFs were involved in immunosuppression in ovarian cancer via upregulation of presenilin 1 (PS1). We took a systemic approach to identify the genes that were highly expressed in CAFs in two independent cohorts of HGSC tumors that had low CTL infiltration, verified how CAFs affect tumor immunosuppression *in vivo* and *in vitro*, and investigated the mechanisms of T-cell trafficking in the TME.

## Materials and Methods

### Human Material, Cell Isolation, and CAF Culture

CAFs were harvested from freshly resected human ovarian cancer and cultured in epidermal growth factor (EGF) containing MCDB105/Medium199 complete media (M-5017, M-6395, and E-4127, Sigma-Aldrich, St. Louis, MO, USA) with 1% penicillin-streptomycin (15,140–122, Invitrogen, Carlsbad, CA, USA) and 10% fetal bovine serum (16,141,079, Gibco, Carlsbad, CA, USA). Mouse fibroblasts from mouse orthotopic ovarian ID8 tumors were isolated and characterized by the following antibodies: fluorescein isothiocyanate (FITC)-conjugated antihuman alpha smooth muscle actin (α-SMA) antibody (ab32575, Abcam, Cambridge, UK), FITC-conjugated anti-immunoglobulin G (anti-IgG) antibody (negative control), and antihuman fibroblast activation protein (FAP) α-antibody (ab28244, Abcam). The cells were isolated enzymatically with collagenase (11,088,785,103, Roche, Mannheim, Germany) in Dulbecco's modified Eagle's medium (11,960,044, Gibco). This research was approved by the Ethical Committee of the First Affiliated Hospital of Soochow University.

### Tumor Growth Assay

Female immunocompetent C57BL/6 mice aged 5–8 weeks were purchased from the Laboratory Animal Center of Soochow University and maintained as described (13). ID8-ip1 (2 × 10^6^) cells were transduced with luciferase-encoding lentivirus and used for C57BL/6 mice. To knock down PS1 expression, we used small interfering RNA (siRNA) + chitosan (150 μg/kg of body weight, 2 times/week, via tail vein). The mice were imaged once a week for bioluminescence signal using a Xenogen IVIS system. At the time of necropsy, the weight, number, and distribution of tumors were recorded. All animal work was performed in accordance with protocols approved by the First Affiliated Hospital of Soochow University.

### Nanoparticles/siRNA Efficiency and Specificity

Nanoparticles/siRNA efficiency and specificity were checked in mouse model. For *in vivo* fluorescence imaging, 100 μl of chitosan/siRNA was intravenously injected into each mouse with tumor sizes of ~100 mm^3^. Then, we used a Maestro EX *in vivo* optical imaging system (Cambridge Research and Instrumentation, Inc.) to carry out the fluorescence imaging at different time points (1, 2, 4, 8, and 24 h). Then, we sacrificed the mice at the point of 24 h and collected the organs (heart, liver, spleen, lung, kidney, and tumor).

### Immunohistochemistry

The cancer samples were labeled for PS1 (NB100-74510, Novus Biologicals, Centennial, CO, USA; PA5-13214, Invitrogen), FAP (ab28244, Abcam), α-SMA (ab32575, Abcam), fibroblast-specific protein (FSP) (ab41532, Abcam), and anti-CD8 (ab85792, Abcam) by immunohistochemistry. Primary antibodies were added to each section. The sections were covered with 4plus biotinylated goat antirabbit IgG (Biocare Medical, Pacheco, CA, USA) and incubated in a humidified chamber for 30 min at room temperature. Primary tumors excised from mouse xenografts were snap frozen for subsequent histological examination. Images were collected using a Leica DFC310 FX microscope (Buffalo Grove, IL, USA). The stained sections were evaluated for the number of CD8+ positive cells using anti-CD8a antibody (550,298, BD Biosciences, Franklin Lakes, NJ, USA). Scoring was divided into the following categories: <5 cells/high-power field (HPF; 40 × magnification), 5–10 cells/HPF, 10–15 cells/HPF, 15–30 cells/HPF, and >30 cells/HPF. The topographic distribution of positive cells was also evaluated. Location relative to the neoplastic follicle (perifollicular, intrafollicular, or neither, i.e., evenly distributed) was recorded.

### Immunofluorescence

Fresh-frozen tissue sections were cut onto Superfrost/Plus slides (Thermo Fischer Scientific, Waltham, MA, USA). A negative control was conducted for each run. For immunofluorescence, cells were grown in eight-well TC Lab-Tek Chamber Slides (Thermo Fischer Scientific). The slides were incubated with the primary antibody overnight at 4 C. Intracellular markers were stained with the primary antibody, followed by antimouse IgG conjugated with Alexa Fluor 488 and/or antirabbit IgG conjugated with Alexa Fluor 594 (Invitrogen). The cell nuclei were stained with 4′,6-diamidino-2-phenylindole (DAPI). Images were recorded using a Leica DFC310 FX microscope.

### Single-Cell Suspension

Single-cell suspensions from primary tumors, lymphoid tissues, and ascites were prepared using Live/Dead fixable dye (eBioscience, San Diego, CA, USA). Ascites were treated by red blood cells lysis buffer (Life Technologies, Carlsbad, CA, USA) for further staining.

### Cell Staining and Flow Cytometry Analysis

Mouse cells were preincubated with purified antimouse CD16/CD32, and human cells were incubated with FcR-blocking reagent (BD Biosciences). Isolated cells were stained with labeled antibodies in phosphate-buffered saline (PBS) with 1% bovine serum albumin (BSA). Dead cells were excluded based on staining with Live/Dead fixable dye (eBioscience). Forkhead box P3 (FOXP3) fixative solution (eBioscience) was used for FOXP3 staining. Prepared samples were analyzed using a flow cytometer (FACSCalibur or FACSAria; BD Biosciences). Data were analyzed using FlowJo software (TreeStar, Ashland, OR, USA). The following beads and antibodies were used: OneComp eBeads (eBioscience), antimouse CD11c Brilliant Violet 421 (BioLegend), antimouse CD11b PE-cy7 (eBioscience), GR1-APC (eBioscience), LY6C-APC-eFluor (eBioscience), F4/80 PE (eBioscience), NK-1.1 Percp-Cy5.5 (eBioscience), anti-CD45 FITC (eBioscience), Live/Dead Aqua (eBioscience), anti-CD4 eFluor 450 (eBioscience), anti-CD8 APC eFluor 780 (eBioscience), anti-CD3 Percp-Cy5.5 (eBioscience), anti-CD19 PE-CY7 (eBioscience), anti-CD25 APC (eBioscience), anti-Foxp3 PE (eBioscience), and anti-CD3 APC (BioLegend).

### Preparation of CAF-Conditioned Medium

CAFs were seeded at 4 × 10^5^ cells per T-75 tissue culture flask, incubated for 24 h at 37°C. New serum-free fibroblast medium (6 ml) was added and treated with PS1 siRNA (10 nM) and control siRNA (10 nM) combined with RNA iMAX reagent (Invitrogen). Four hours later, the medium was changed with complete fibroblast medium with 10% fetal bovine serum (FBS). The conditioned medium was collected between days 2 and 3. An Amicon Ultra-15 Centrifugal Filter Unit (Merck Millipore, Burlington, MA, USA) was used to concentrate the conditioned medium for analysis.

### Lymphocyte Proliferation Assays

T-cell proliferation was assessed using the carboxyfluorescein succinimidyl ester dilution assay. Cultured mouse splenocytes were sorted by magnetic-activated cell sorting beads labeled with antimouse CD4 (130-049-201, Miltenyi Biotec, Bergisch Gladbach, Germany) and antimouse CD8 (130-049-401, Miltenyi Biotec). Latex beads (5 μm in diameter, 4%; Invitrogen) were coated with various concentrations of anti-CD3 (BD Bioscience; Cat: 553,058) and anti-CD28 (BE0015-5; BIO X cell) to activate T cells in the absence of accessory cells. Sorted cells washed in PBS by centrifugation at 400 × *g* for 4 min and resuspended in 0.5 ml of PBSA (0.2%) and carboxyfluorescein diacetate succinimidyl ester (CFSE) (Cat. No. C34554 Molecular Probes, Life Technologies, CA, USA) at 5 μg/ml, followed by incubation at 37°C for 15 min, and immediate cooling on wet ice and dilution in 10 ml of cold Iscove's modified Dulbecco's medium (IMDM). CFSE-labeled cells were added at a density of 1–5 × 10^5^ live cells per well. Immediately after initiation of cocultures, cells were mixed with conditional media from PS1 siRNA-treated CAFs (2:1 ratio) with different concentrations. Mixed cultures were incubated for 4–5 days at 37°C. Following incubations, lymphocytes were harvested, centrifuged at 400 × *g* for 4 min, and resuspended in 0.5 ml PBS. CFSE fluorescence was analyzed on a FACS Calibur™ (Becton Dickinson) flow cytometer, and flow cytometric data were analyzed by FlowJo (TreeStar, Ashland, OR, USA) software. To examine the function of PS1 siRNA to CD8+ T-cell activation, CD107a (EBioscience) used to measure cytolytic activity of CTL and intracellularly stained with IFN-g-FITC (Miltenyi Biotec). Two million five hundred thousand events were collected during flow cytometric analysis. Data acquired utilizing a Fortessa LSRIII supported by FACS diva software (BD Biosciences, San Jose, CA, USA) and analyzed using the FlowJo 10.0.8 software (Ashland, OR, USA).

### Migration Assays

For chemotactic migration assays, Transwell membrane inserts (6.5 mm, 5 μm pores; Corning, Corning, NY, USA) were coated with 10 μg/ml fibronectin (Sigma-Aldrich) for 2 h at 37°C. Afterwards, 100 μl purified suspension of mouse lymphocytes (containing 5 × 10^5^ cells) was added to the top chamber. The lower chambers were filled with 600 μl fresh fibroblast growth medium at a 1:1 ratio with conditioned medium from CAF culture and 0.5 μg/ml CCL19 (R&D Systems, Minneapolis, MN, USA) was used as positive control. After 4 h of incubation at 37°C in an atmosphere of 5% CO_2_, all lymphocytes invading the lower chamber were harvested. Total cell and viable cell counts were determined using a hemocytometer.

### Real-Time PCR

Total RNA was isolated using TRIzol (Invitrogen). We synthesized complementary DNA (cDNA) with a Superscript III Platinum One-Step qPCR kit (Invitrogen). Subsequent experiments were conducted using an ABI Prism 7,500 (Applied Biosystems, Beverly Hills, CA, USA), and gene expression levels were normalized against the housekeeping gene β-actin (Invitrogen).

### Bioinformatics Analysis

Ingenuity Pathway Analysis (IPA, QIAGEN, Redwood City, CA, USA) software was used for pathway analysis https://www.qiagenbioinformatics.com/products/ingenuity-pathway-analysis/).

### Statistical Analysis

Each value is the average of the six samples, and groups were compared using unequal variances two-tailed Student's *t*-test. Each experiment was repeated at least three times, and a representative experiment is presented. Data were analyzed using SPSS v.19.0 software (SPSS Inc., Chicago, IL, USA) and were shown as mean ± SD. A *p* < 0.05 was considered statistically significant.

## Results

### High Expression of PS1 in CAFs May Result in Low CD8+ T Cell Counts in the TME

**Figure 1:**
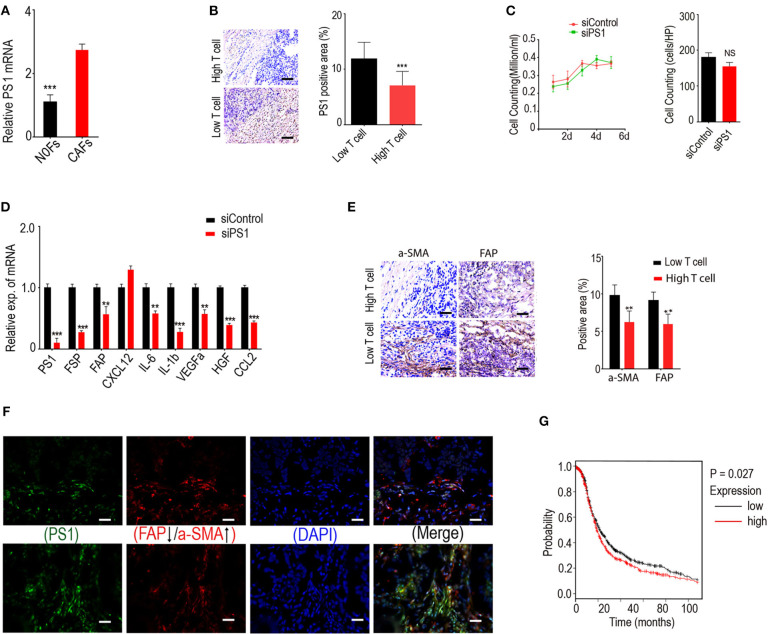
Low tumor-infiltrating lymphocyte counts in ovarian tumor coincide with activated CAFs due to highly expressed presenilin 1 (PS1). **(A)** Expression of PS1 in CAFs and NOFs by quantitative PCR (qPCR). CAFs, cancer-associated fibroblasts; NOFs, normal ovarian fibroblasts (*n* = 5). ***p < 0.001. **(B)** Expression of PS1 is strongly correlated with tumor-infiltrating T lymphocytes. Results were means SD of triplicate samples from three representative experiments. Scale bar = 50 μm. ***p < 0.001. **(C)** Cell growth after patient-derived cancer-associated fibroblasts silenced by small interfering RNA (siRNA). Results obtained after 6 days culture (*n* = 3). **(D)** Expression of PS1, fibroblast-specific protein (FSP), fibroblast activation protein (FAP), and several immunosuppressive factors checked by qPCR after silencing PS1 in patient-derived fibroblasts. **p < 0.01, ***p < 0.001. **(E)** CD8+ cytotoxic T lymphocyte (CTL) density in human ovarian tumor samples checked by immunohistochemistry and its association with the expression of two CAF markers alpha smooth muscle actin (α-SMA) and FAP. Scale bar = 50 μm. **p < 0.01. **(F)** PS1 colocalized with different CAF markers in human ovarian tumors. Scale bar = 50 μm. **(G)** Kaplan–Meier plot shows PS1 is correlated with poor prognosis in ovarian cancer patients. Error bars denote mean ± SEM (standard error of the mean). *p < 0.05; **p < 0.01; ***p < 0.005; ns, statistically not significant. Statistical analyses were performed using one-way ANOVA with Tukey multiple comparison test.

We started our study from a candidate pool with nine differential expressed genes (>8-fold) related to CTL penetration regulation, which were obtained from microarray assay with fibroblast samples of 10 normal human ovaries and 83 ovarian tumors. These genes were *ALDCA, ACKR3, BACE2, ATP5C1, ARF4, CCT3, CKAP4, ATP1B1*, and *ARPC3*, and they were all significantly associated with lower CD8+ T cell counts in HGSC and higher CD8+ T cell numbers in CAFs when compared with normal fibroblasts. To identify specific genes that may play important roles in the differential expression of the nine genes, we conducted bioinformatics analyses using Ingenuity Pathway Analysis software and identified PS1 as a common upstream regulator for three of these nine genes: *ATP1B1, ARF4*, and *ATP5C1*. Using freshly isolated human normal ovarian fibroblasts (NOFs) and cancer-associated fibroblasts (CAFs) derived from HGSC, we confirmed that PS1 expression was indeed higher in CAFs when compared with NOFs (Figure 1A).

To investigate the distribution of CD8+ tumor-infiltrating lymphocytes, we performed immunohistochemistry using clinical samples. T cells were counted in ovarian tumors according to CD8+ counts/HPF to identify tumors with high and low T cell counts. Immunohistochemistry for PS1 was also conducted. We found that low tumor-infiltrating lymphocyte counts in ovarian cancer were strongly correlated with high expression of PS1 (Figure 1B). Notably, silencing of PS1 in patient-derived CAFs using siRNAs did not affect the cell growth (Figure 1C) (*p* > 0.05) but significantly decreased the expression of several immunosuppressive factors as well as FAP and fibroblast-specific protein 1 (FSP1, also known as S100A4), two important makers for CAFs (Figure 1D) (*p* < 0.05). To examine whether fibroblast activation in ovarian tumors is related to lower tumor-infiltrating lymphocyte counts in tumor parenchyma, we evaluated two different fibroblast activation markers, FAP and α-SMA. Fifty-six human ovarian tumor samples checked by immunohistochemistry showed that CD8+ CTL density is significantly decreased in patients with a poor survival rate and associated with significantly higher expression of CAF markers (Figure 1E). Next, we identified that PS1 colocalized with different CAF markers in a set of human ovarian tumors (Figure 1F). In line with our data, PS1 was correlated with poor prognosis in ovarian cancer patients in the Kaplan–Meier plot (Figure 1G). Taken together, these results suggest that high expression of PS1 in CAFs may result in low CD8+ T cell counts in the TME.

### Silencing PS1 in Ovarian Tumors Results in Enhanced CTL Penetration and Reduced Tumor Burden *in vivo*

Nanoparticles could effectively accumulate in the tumor site of these ID8 tumor-bearing mice, and the fluorescence signal increased with time and reached the peak at 24 h (Supplemental Figure 1). We gave silenced PS1 in different cancer cell lines; however, there were no significant changes in cytokine releasing. We treated mice cancer cell line (ID-8) with nanoparticles/PS-1siRNA before *in vivo* study. There were no significant growth ratio differences between the two groups' cell growth curve (Supplemental Figure 1).

**Figure 2:**
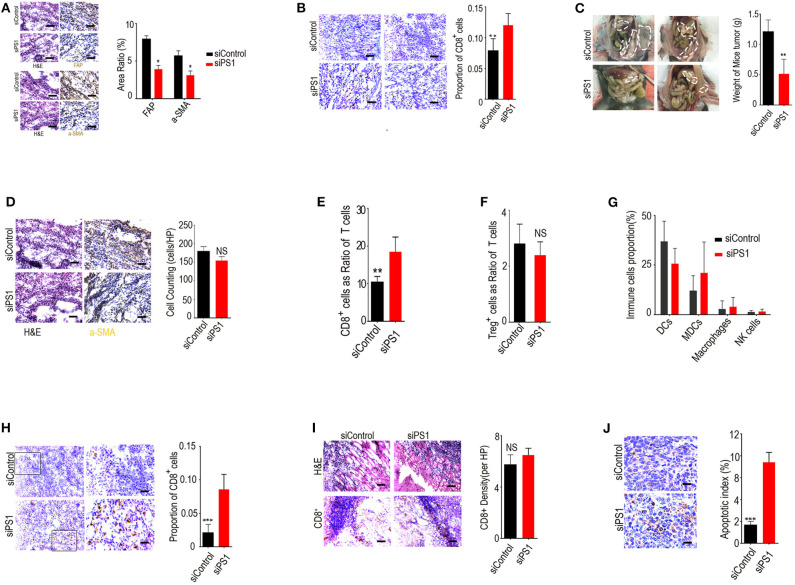
Silencing presenilin 1 (PS1) in ovarian tumors results in enhanced cytotoxic T lymphocyte (CTL) penetration and reduced tumor burden *in vivo*. **(A)** PS1, α-SMA, and FAP expression in the stromal compartment of the tumors after treatment with chitosan nanoparticles/PS1 siRNA. Results are representative of data generated in two independent experiments. *p < 0.05. **(B)** Tumor-infiltrating CD8+ CTLs after treatment with chitosan nanoparticles/PS1 in C57B/6 mice bearing orthotopic ID8 ovarian tumors. The result is from one of three similar experiments (*n* = 3). **(C)** Tumor burden in mice after 3 weeks treated with chitosan nanoparticles/PS1 small interfering RNA (siRNA). **(D)** Cancer-associated fibroblast (CAF) counts in juxtatumoral tissues by HE staining after siRNA treatment (*n* = 3). **(E–G)** The number of CD8+ T cells, dendritic cells, myeloid-derived suppressor cells, nature killer cells, and macrophages in tumors compared to the controls, checked by flow cytometry (*n* = 3). **(H)** CD8+ T cell density in the tumor parenchyma by immunohistochemistry after treatment with PS1 siRNA (*n* = 3). **(I)** CD8+ T-cell density in the in the tumor stroma by immunohistochemistry after treatment with PS1 siRNA. **(J)** Caspase-3 expression in the tumor parenchyma densely infiltrated by CD8+ T cells than in areas sparsely infiltrated by CD8+ T cells in PS1 siRNA-treated mouse tumors. The result is from one of three similar experiments (*n* = 3) (**A**/**B**/**D**/**H**/**I**/**J** scale bar = 50 μm). **P < 0.01 and ***P < 0.005.

To assess the effect of silencing tumoral expression of PS1 *in vivo*, we systemically delivered murine PS1 siRNA into C57B/6 mice bearing orthotopic ID8 ovarian tumors using chitosan nanoparticles. Treatment with chitosan nanoparticles/PS1 siRNA resulted in significant downregulation of PS1 expression in stromal compartment of the tumors and a decrease in the expression of both α-SMA and FAP (Figure 2A) (*p* < 0.05). This ultimately led to relief from the immunosuppressive networks in these tumors and markedly increased the number of tumor-infiltrating CD8+ CTLs (>12-fold increase, *p* < 0.05, Figure 2B). Significant reduction in tumor burden was observed in mice treated with chitosan nanoparticles/PS1 siRNA compared to the group treated with chitosan nanoparticles/control siRNA following 3 weeks of therapy (Figure 2C) (*p* < 0.05). Interestingly, the CAF counts in juxtatumoral tissues were not dramatically decreased by siRNA treatment (Figure 2D).

We examined the population of immune cells in the mouse tumors by flow cytometry and found that the number of CD8+ T cells was significantly increased in tumors compared with controls, whereas the numbers of dendritic cells, myeloid-derived suppressor cells, nature killer cells, and macrophages were not significantly changed after treatment (Figures 2E–**G**) (*p* > 0.05).

We next assessed the distribution of CD8+ T cells in the tumor stroma and parenchyma by immunohistochemical staining. Our results revealed that CD8+ T-cell density was significantly increased in tumor parenchyma but not in tumor stroma after treatment with PS1 siRNA (Figures 2H,I). To determine whether CD8+ T cells were also involved in PS1 siRNA-mediated antitumor effect, we checked the apoptosis rate in these tumors. Caspase-3 expression was higher in the tumor parenchyma densely infiltrated by CD8+ T cells than in the areas sparsely infiltrated by CD8+ T cells, which was observed in PS1 siRNA-treated mouse tumors (Figure 2J). These results indicated that high expression of PS1 in CAFs was strongly correlated to reduced infiltration of CD8+ T cells into the tumor parenchyma.

### PS1 Silencing Enhances Dendritic Cell Function, T-Cell Migration, and Proliferation *in vitro*

**Figure 3:**
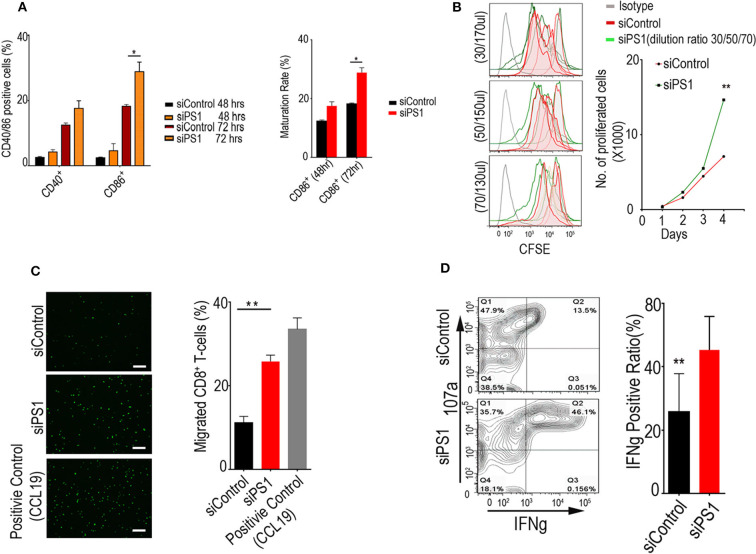
Presenilin 1 (PS1) silencing enhances dendritic cell function, T-cell migration, and proliferation *in vitro*. **(A)** Dendritic cells' ability to present tumor antigens to other effector cell types after incubation with PS1 small interfering RNA (siRNA)-treated cancer-associated fibroblast (CAF)-conditioned medium. Cells accounted for CD40+/CD86+ ratio were checked by flow cytometry (*n* = 3), *p < 0.05. **(B)** T-cell proliferation potential after incubation of CD8+ T cells with PS1 siRNA-treated CAF-conditioned medium. Carboxyfluorescein diacetate succinimidyl ester (CFSE)-labeled lymphocytes have been used to analyze the relationship between cell division and differentiation of cell function. Different dilution ratio of conditioned media were checked within 4 days by flow cytometry (30/50/70 μl, 200 μl total volume) (*n* = 5). **(C)** The conditioned media collected from PS1 siRNA-treated CAFs increased T-cell migration markedly. CCL19 as the positive group (*n* = 3). Scale bar = 50 μm. **p < 0.01. **(D)** Cytotoxic T-cell activity after PS1 siRNA treatment. Effects of increasing 107a and interferon gamma (IFN-γ) secretion measured (*n* = 3), **p < 0.01. Error bars denote mean ± SEM. Statistical analyses were performed using one-way ANOVA with Tukey multiple comparison test.

Given the ability of PS1 to regulate multiple proinflammatory and immunosuppressive molecules, we assessed whether PS1 downregulation in CAFs had an impact on migration or the function of immune cells, with focus on CTLs and dendritic cells. CD8+ T cells were isolated from C57B/6 mouse-derived splenocytes, and dendritic cells were generated by incubating bone-marrow-derived cells with FLT3LG. After migration assay, we exposed the dendritic cells and T cells to conditioned media collected from CAFs treated with control siRNA or PS1 siRNA (48 h post-transfection). Incubation with PS1 siRNA-treated CAF-conditioned medium resulted in a significant increase in CD40 and CD86 levels in dendritic cells (*p* < 0.05), indicating an increase in their ability to present tumor antigens to other effector cell types (Figure 3A). Incubation of CD8+ T cells with the same conditioned medium resulted in enhanced T-cell proliferation potential (Figure 3B). When the conditioned media collected from PS1 siRNA-treated CAFs were put in the lower compartment of migration chambers, there was a 2-fold increase in the ability of T cells to migrate across the endothelial monolayer (Figure 3C). Importantly, PS1 siRNA treatment resulted in substantial enhancement in cytotoxic T-cell activity, as measured by interferon gamma (IFN-γ) (Figure 3D).

### PS1 Enhances IL-1β Production via WNT/β-Catenin Pathway in CAFs

**Figure 4:**
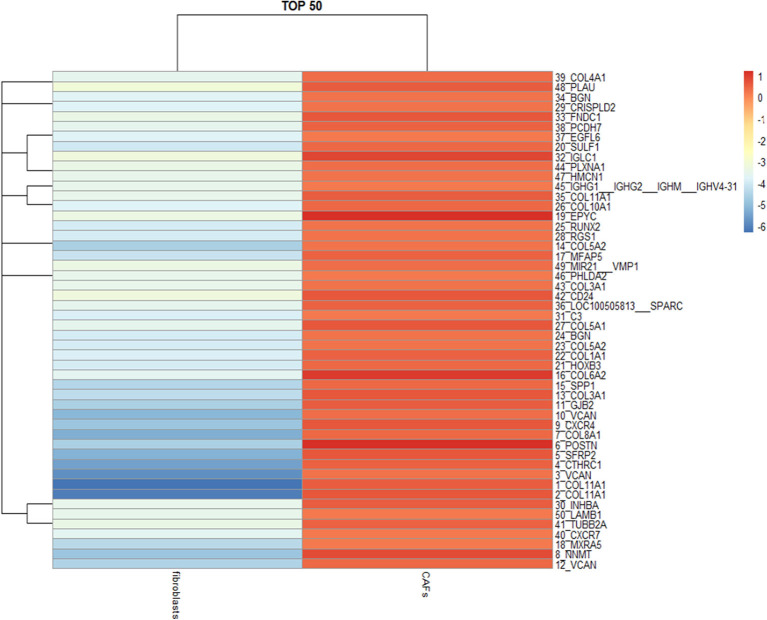
Ingenuity Pathway Analysis reveals immune cell trafficking as one of the top pathways affected by presenilin 1 (PS1) small interfering RNA (siRNA).

**Figure 5:**
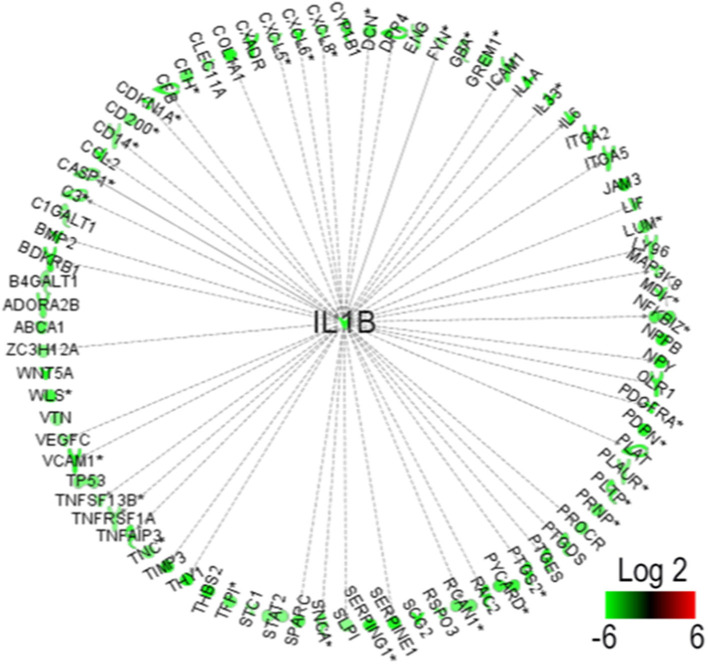
Interferon-1β (IL-1β) identified as the key molecule upstream downregulated by presenilin 1 (PS1) small interfering RNA (siRNA).

**Figure 6:**
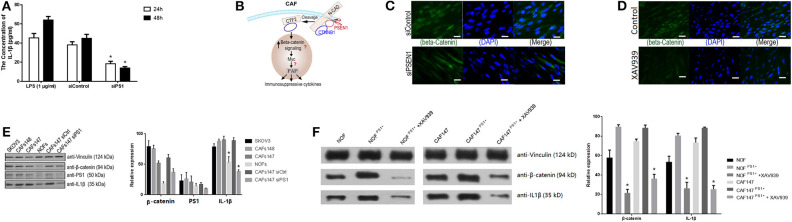
Presenilin 1 (PS1) enhances interferon-1β (IL-1β) production via WNT/β-catenin pathway in cancer-associated fibroblasts (CAFs). **(A)** IL-1β levels in the conditioned media of PS1 small interfering RNA (siRNA)-treated CAFs and control checked by ELISA. Lipopolysaccharide (LPS) (1 μg/ml) as the positive control. The result is from one of three similar experiments (*n* = 5). *p < 0.05. (**B)** Ingenuity Pathway Analysis identified β-catenin in mediating the effect of PS1 on IL-1β. **(C)** Silencing PS1 in CAFs decreased β-catenin levels, and its subcellular location was evaluated by immunofluorescence staining. β-Catenin was expressed in cytoplasm of CAFs (green). (*n* = 5). Scale bar = 5 μm. **(D)** β-Catenin levels in the nucleus in XAV939-treated CAFs were evaluated by immunofluorescence staining. XAV939 as the chemical inhibitor to β-catenin (*n* = 5). Scale bar = 5 μm. **(E)** β-Catenin levels in cells were assessed by Western blot after treated by siPS1. The protein levels of PS1 and IL-1β in different cell lines were checked. Ovarian cancer cell SKOV3 and two CAFs cell lines: CAF147/CAF148 was checked. Vinculin was used as loading controls, respectively. NOF, normal fibroblasts; CAFs, cancer-associated fibroblasts. *p < 0.05. **(F)** β-Catenin and IL-1β checked by Western blot in NOFs and CAFs when treated with XAV939. PS1 was overexpressed in CAFs and NOFs by transfected PS1 short-hair RNA (shRNA), which combined with XAV939 treatment. NOF, normal fibroblasts; CAFs, cancer-associated fibroblasts. Error bars denote mean ± SEM. *p < 0.05. Statistical analyses were performed using one-way ANOVA with Tukey multiple comparison test.

To further investigate the molecular mechanism by which PS1 regulates inflammatory networks in CAFs, genomic analysis was performed using human HGSC-derived CAFs treated with control siRNA or PS1 siRNA. Pathway enrichment analysis revealed immune cell trafficking to be one of the top pathways affected by PS1 siRNA (*p* < 5 × 10^−6^) (Figure 4). Interleukin (IL)-1β was identified as the key molecule upstream of a large panel of immunosuppressive factors that are downregulated following PS1 silencing (Figure 5). We then examined conditioned media of PS1 siRNA-treated CAFs by ELISA and found that, compared with media of control siRNA-treated CAFs, IL-1β levels were significantly decreased in the conditioned media of PS1 siRNA-treated CAFs (Figure 6A) (*p* < 0.05). Further analysis using the Ingenuity Pathway Analysis software indicated the potential role of β-catenin (CTNNB1) in mediating the effect of PS1 on IL-1β (Figure 6B). After silencing PS1 in CAFs, changes in β-catenin levels and its subcellular location were evaluated by immunofluorescence staining (Figure 6C). In PS1 siRNA-treated CAFs, β-catenin levels decreased in the nucleus (Figure 6D). β-Catenin levels in the cytosolic and nuclear fractions were checked by Western blotting. In addition, β-catenin activity in cells was assessed with Western blotting (Figure 6E). To confirm whether the nuclear translocation of β-catenin was critical for IL-1β activation, we transfected CAFs with PS1-expressing plasmids using XAV939 to inhibit the translocation of β-catenin into the nucleus and assess IL-1β levels. PS1 enhanced the nuclear β-catenin expression in CAFs and was accompanied by high IL-1β levels, which could be inhibited by XAV939 (Figure 6F) (*p* < 0.05), indicating that PS1 regulates IL-1β via β-catenin activation.

## Discussion

Our current study explored the underlying mechanisms of how CAFs affect immunosuppression in the TME. *In vitro* and *in vivo* experiments both showed that CAFs suppress the TME via high expression of PS1. PS1 silencing led to enhanced CTL infiltration and reduced tumor size. Enhanced dendritic cell function and T-cell migration were also strongly associated with PS1 silencing, indicating that CAFs block the migration of tumor-infiltrating lymphocytes into the tumor via PS1. IL-1β was identified as the downstream molecule regulated by PS1, which acts via the WNT/β-catenin pathway.

It was reported that PS1 activated the epidermal growth factor receptor (EGFR)–signal transducers and activators of transcription (STAT) pathway to inhibit apoptosis in head and neck squamous cell carcinoma (14). In esophageal and bladder cancer, PS1 was involved in resistance to chemotherapy drugs (15). In breast cancer, PS1 could affect cell invasion and migration by modulating E-cadherin and COX-2 (16). Our study revealed that CAFs were activated to suppress the TME via high expression of PS1, indicating that CAF activity could be normalized by inhibiting PS1. Furthermore, silencing PS1 did not affect the cell growth. PS1 was also found to colocalize with α-SMA and FAP in human ovarian cancer *in vivo*, which suggested that PS1 was located in the nucleus and secreted by CAFs.

We found that high expression of PS1 suppressed the distribution of tumor-infiltrating lymphocytes. The density of CD8^+^ CTLs in human ovarian cancer was negatively correlated with the expression of PS1, suggesting that PS1 might serve as a prognostic biomarker. Tumor-cell-stimulated CAFs released PS1 and affected the infiltration of CD8^+^ T cells, the numbers of which were significantly decreased in patients with poor survival rates. A recent research (17) showed that, in intratumoral esophageal cancer tissues, CD8^+^ tumor-infiltrating lymphocytes were negatively correlated with CAFs. IL-6 was highly expressed and increased colon tumor growth. There were fewer CD8^+^ tumor-infiltrating lymphocytes in untreated tumors, suggesting that CAFs regulate immunosuppressive tumor-infiltrating lymphocyte populations in the TME via IL-6. Silencing PS1 in ovarian tumors results in a significant increase in CTLs in preclinical models of HGSC. Silencing PS1 in CAFs also results in an enhancement of T-cell activity. When PS1 siRNA is delivered, it can synergistically impact the therapeutic efficacy of existing immunomodulating agents in HGSC and pancreatic ductal adenocarcinoma tumors, leading to induction of potent tumor-antigen-specific immune responses. In our study, PS1 silencing leads to increased numbers of CD8^+^ tumor-infiltrating lymphocytes and is negatively correlated with prognosis, and CAFs affect intratumoral CD8^+^ tumor-infiltrating lymphocytes in the TME.

Tumor cells communicate with fibroblasts and stromal cells in the TME via cytokines and chemokines. IL-1 is upregulated in many tumor types and has been implicated in tumor progression (18). IL-1β is abundant at tumor sites, indicating that it is involved in the process of carcinogenesis, tumor growth, and invasiveness, and the patterns of tumor–host interactions (19, 20). We found that IL-1β was the key molecule upstream of immunosuppressive factors. Silencing PS1 significantly decreased the expression of IL-1β, suggesting that blocking PS1 might reverse the immunosuppression in ovarian cancer. Immunofluorescence staining for β-catenin showed decreased expression levels in the nucleus and cytosol after PS1 silencing in CAFs, suggesting that PS1 affected IL-1β secretion via β-catenin. It was verified by transfecting CAFs with PS1-expressing plasmids and the β-catenin inhibitor XAV939 *in vitro*. Moreover, our findings are corroborated in mouse breast cancer (20).

There were also several limitations in this study. First, although we evaluated the localization of CD8^+^ T cells, the mechanism behind it were not elucidated. Second, although we verified that PS1 enhances IL-1β production via the WNT/β-catenin pathway in CAFs, we did not clarify how IL-1β affects CTL proliferation and function. We conducted bioinformatics analysis by Ingenuity Pathway Analysis and found that IL-1β affects CTL proliferation and function via the endothelial PAS domain-containing protein 1 (EPAS1)–inducible nitric oxide synthase (iNOS)–nitric oxide (NO) pathway. Future studies ought to focus on this pathway and elucidate the mechanism.

## Conclusions

In conclusion, we demonstrated that CAF-derived PS1 modulates immunosuppression in the TME via the WNT/β-catenin pathway. Our findings showed an actionable way to reverse the immunosuppression of highly expressed PS1 in CAFs by blocking PS1 to improve CTL trafficking and migration and therefore improve the therapeutic outcome in different types of cancers, including ovarian cancer, either independently or in combination with immune checkpoint inhibitors such as PD-1 or PD-L1 blockers.

## Data Availability Statement

The datasets used and/or analyzed during the current study are available from the corresponding author on reasonable request.

## Ethics Statement

The animal study was reviewed and approved by the Ethical Committee of the First Affiliated Hospital of Soochow University.

## Author Contributions

FS, HZ, YC, and XZ conceived the project and designed the experiments. FS, JZ, JW, YX, and RJ participated in *in vitro* experiments. HZ performed computational analyses. YG designed and performed nanostring analysis. FF prepared viral particles. YS and GZ performed microarray analysis. FS, LF, HZ, and YC participated in *in vivo* experiments. YC, XZ, HZ, and FS analyzed the clinical data. FS, HZ, and YC analyzed all the data and wrote the paper. All authors edited and approved the final manuscript.

## Conflict of Interest

The authors declare that the research was conducted in the absence of any commercial or financial relationships that could be construed as a potential conflict of interest.

## Supplementary Material

Supplementary Figure 1 **(A)** Showed as nanoparticles/siRNA injected into the mice through tail vein after 24H by time-lapsed NIR fluorescence *in vivo* imaging. **(B)** Maestro EX *in vivo* optical imaging system (Cambridge Research and Instrumentation, Inc.) to carry out the fluorescence imaging at 24 hours after injection of nanoparticles/siRNA. The tumor and liver has the top enrichment of nanoparticles. Abbreviations: Tu, Tumor; Lu, Lung; Sp, Spleen; Ki, Kidney; Li, Liver; He, Heart. **(C)** Silencing PS1 in different cancer cell lines by PS1 siRNA, however, there were no significant changes in cytokines releasing in mRNA level. **(D)** Treated mice cancer cell line (ID-8) with nanoparticles/PS-1siRNA before *in vivo* study. There were no significant growth ratio difference between in two groups cell growth curve.

## Abbreviations

CAFs: cancer-associated fibroblasts
TME: tumor microenvironment
CTLs: cytotoxic T lymphocytes
HGSC: high-grade serous ovarian carcinoma.
