# Supplementary figures and images for: CTL Attenuation Regulated by PS1 in Cancer-Associated Fibroblast
Source: Front Immunol. 2020 Jun 10;11:999. doi: 10.3389/fimmu.2020.00999 (PMC7297945; doi:10.3389/fimmu.2020.00999)

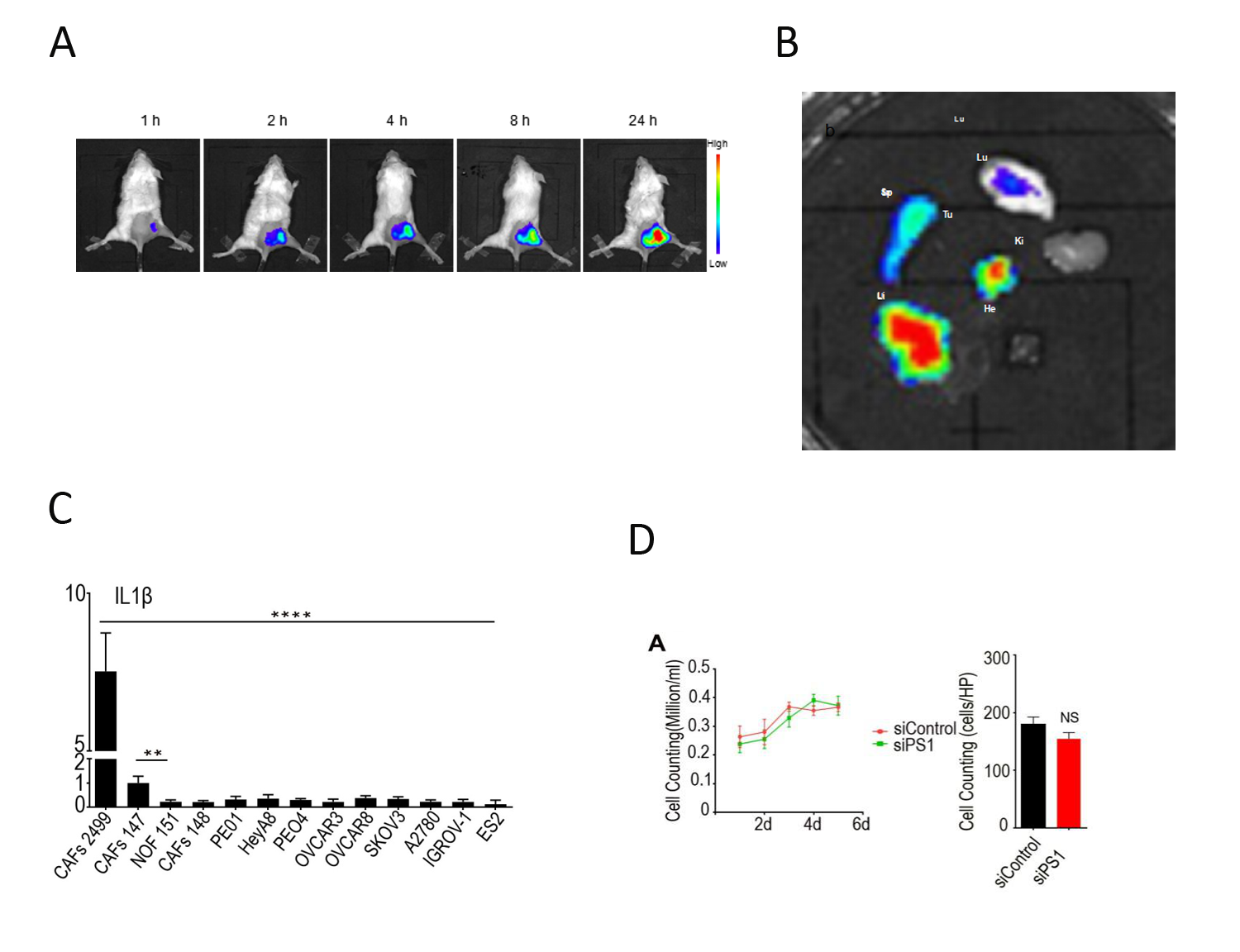

Supplement: Supplementary Figure 1 — (A) Showed as nanoparticles/siRNA injected into the mice through tail vein after 24H by time-lapsed NIR fluorescence in vivo imaging. (B) Maestro EX in vivo optical imaging system (Cambridge Research and Instrumentation, Inc.) to carry out the fluorescence imaging at 24 hours after injection of nanoparticles/siRNA. The tumor and liver has the top enrichment of nanoparticles. Abbreviations: Tu, Tumor; Lu, Lung; Sp, Spleen; Ki, Kidney; Li, Liver; He, Heart. (C) Silencing PS1 in different cancer cell lines by PS1 siRNA, however, there were no significant changes in cytokines releasing in mRNA level. (D) Treated mice cancer cell line (ID-8) with nanoparticles/PS-1siRNA before in vivo study. There were no significant growth ratio difference between in two groups cell growth curve. [file Image_1.TIF]
